# Tryptase Positivity in Chronic Myeloid Leukemia With Marked Basophilia

**DOI:** 10.7759/cureus.9577

**Published:** 2020-08-05

**Authors:** Youssef Khafateh, Barina Aqil

**Affiliations:** 1 Pathology and Laboratory Medicine, University of Louisville, Louisville, USA; 2 Pathology, University of Louisville/Northwestern University, Louisville, USA

**Keywords:** chronic myeloid leukemia, basophilia, tryptase

## Abstract

Chronic myeloid leukemia (CML) is the most common chronic myeloproliferative disorder, which was the first to be described and understood at a molecular level. Marked basophilia can be seen in CML and other neoplastic and reactive processes. Tryptase is a serine protease that is mainly expressed in mast cells, whereas basophils express only trace amounts of the enzyme. Therefore, it has always been regarded as a specific marker for mast cells.

We report a case of a 41-year-old male who had been diagnosed with CML eight years ago, and, interestingly, his most recent bone marrow biopsy demonstrated an accelerated phase of the disease with a significant increase of basophils count. These basophils were immunoreactive with tryptase along with CD123.

In the literature, this phenomenon of tryptase immunoreactivity by basophils has been described in association with CML, primary myelofibrosis, and myelodysplastic syndrome. Therefore, our finding supports these data and suggests that tryptase should not be regarded as a specific marker for mast cells when approaching various myeloid neoplasms including CML.

## Introduction

Chronic myeloid leukemia (CML) is a myeloproliferative neoplasm (MPN) in which granulocytes are the major proliferative component [1]. CML is the most common disorder of this category, which was the first to be described and understood at a molecular level. It arises from an abnormal pluripotent hematopoietic stem cell and can occur at any age, with peak incidence between 50 and 60 years of age [1]. The natural history of CML has three distinct phases: a chronic phase (CP) followed by an accelerated phase (AP) and then, finally, a blast phase (BP) with a very poor outcome [2]. The association between increased basophils and MPNs is a well-known feature, and basophilia has been regarded as an unfavorable sign of CML since the 1970s. While basophilia is seen in a variety of reactive and other neoplastic conditions, marked basophilia in association with CML is notably seen in the AP (>20%) or during blast crisis [3,4].

Basophils and mast cells are inflammatory cells that are implicated in allergic reactions as they express high-affinity IgE (immunoglobulin E)-binding sites and various granular mediators [5]. Tryptase is a serine protease primarily expressed and produced from mast cells [6]. It has been regarded as a clinical indicator of mast cell dependent events, mainly in systemic allergic conditions, and has a diagnostic value as an immunohistochemical stain in patients with mastocytosis [7,8]. Small amounts of tryptase are found to be in basophils but not in other leukocytes. Tryptase quantities in basophils were less than 1% of those in tissue mast cells [9]. Basophils share many features with mast cells; however, recent work has shown unique roles and functions of basophils [10]. Interestingly, some studies have raised the possibility that neoplastic basophils can express a significant amount of tryptase [11,12].

## Case presentation

We report a case of a 41-year-old male who initially presented with leukocytosis and splenomegaly eight years ago and was diagnosed with CML, BCR/ABL1 positive. The bone marrow examination showed hypercellular marrow (>90% cellular) with granulocytic hyperplasia and no increase in blasts. Cytogenetic analysis demonstrated the presence of the Philadelphia chromosome t(9;22), establishing the diagnosis of CP CML. The patient was subsequently lost to follow-up for approximately five years.

The patient presented in 2015 with a three-month history of weight loss and abdominal distension. The complete blood count revealed anemia, thrombocytopenia, and leukocytosis, with peripheral blood fluorescence in situ hybridization (FISH) positive for the BCR/ABL1 fusion. He was restarted on dasatinib, which he continued for several months until he developed dasatinib-related pancytopenia, which required RBC transfusion. The bone marrow biopsy was performed and showed continuous involvement by CML in the CP with mild reticulin fibrosis.

**Figure 1:**
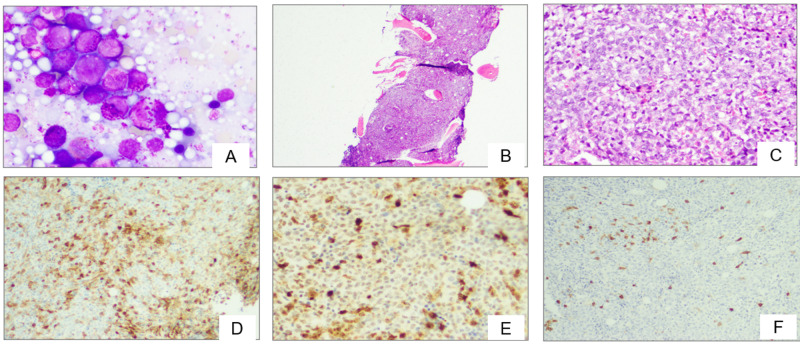
(A) Bone marrow touch preparation with increased basophils. (B and C) Hypercellular core with numerous atypical mononuclear cells, which are positive for CD123 (D) and tryptase (E). (F) CD117 highlights few scattered mast cells.

In 2017, while the patient was admitted to the hospital with a fever, bone marrow biopsy was performed due to significantly increasing WBC counts, reaching >150 × 10^9^/L. Biopsy revealed markedly hypercellular marrow (>90%) with myeloid hyperplasia and left-shifted maturation. Marked basophilia was noted in peripheral blood and bone marrow, reaching up to 80% of leukocytes in blood. Blasts were increased, comprised approximately 3-10% of marrow cellularity, as highlighted by the CD34 immunohistochemistry. Interestingly, tryptase was positive in some of the mononuclear cells on bone marrow biopsy. These cells were also positive for CD123, confirming that they were basophils. In contrast, CD117 highlighted only scattered mast cells (Figure 1).

**Figure 2:**
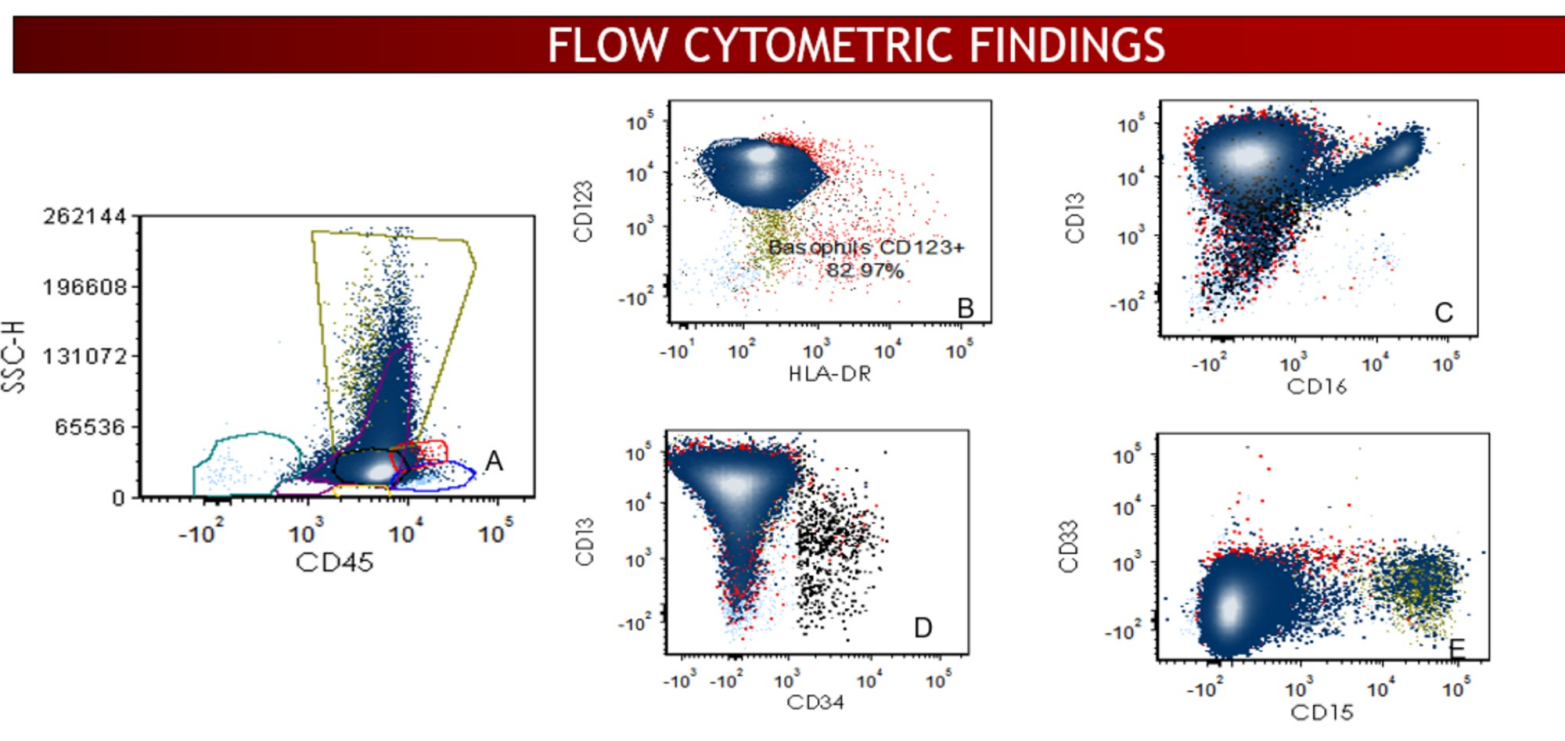
(A-E) Flow cytometric analysis detected increased basophils (CD123+) with normal immunophenotypic profile.

Flow cytometric analysis of the bone marrow revealed an abnormal population of myeloid blasts. In addition, the basophils were found to be markedly increased, comprising approximately 80% of total cells (Figure 2). These findings were morphologically and immunophenotypically consistent with an AP of CML.

**Figure 3:**
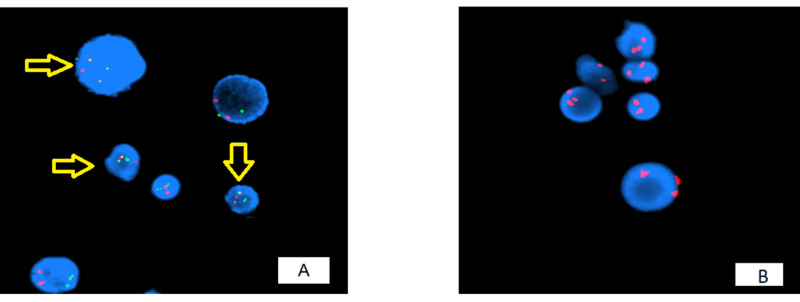
(A) BCR/ABL1 fusion: red and green signals correlate with chromosomes 9 and 22, whereas yellow signals, arrows, indicate translocation (fusion). (B) Trisomy 8 detected by FISH analysis, where three copies (each indicated by a red dot) of chromosome 8 are seen in most of the cells. FISH, fluorescence in situ hybridization

The concurrent FISH analysis on bone marrow aspirate detected BCR/ABL1 fusion as well as trisomy 8, which is another defining criterion for the AP of the disease (Figure 3).

Later that month, due to symptomatic splenomegaly, the patient underwent splenectomy. The spleen was severely enlarged (2,900 grams) and showed involvement by CML. Markedly increased basophils (about 57% of total cells) were detected by flow cytometric analysis, similar to the bone marrow findings.

## Discussion

Basophils and mast cells are inflammatory cells that are found in virtually all human tissues and are involved in the pathogenesis of various allergic reactions. By releasing a variety of chemical mediators, they could also play a role in the pathophysiology of a wide range of inflammatory disorders [5]. Both human mast cells and basophils arise from CD34+ human progenitor cells. Although these two cell types share many features, several differences have also been observed.

Mast cells are tissue resident inflammatory cells that respond to innate and adaptive immunity signals and located primarily in association with blood vessels and epithelial surfaces [5]. Mast cells are involved not only in the pathogenesis of hypersensitivity disorders but also in neoplastic conditions such as mastocytosis. Two major subtypes of mast cells exist based on the presence of tryptase only (MCT cells) or tryptase with mast cell specific chymase (MCTC cells). In general, mast cells express KIT (CD117), which is a receptor for stem cell factor.

Mast cell tryptase is a tetrameric neutral serine protease with a molecular weight of 134 kDa. The genes that encode mast cell tryptase are located on the short arm of chromosome 16. The enzyme is made up of four non‐covalently bound subunits, and each subunit has one active enzyme site [6]. The release of tryptase from activated mast cells may stimulate secretion from neighboring mast cells, thus providing an amplification signal [6]. High tryptase levels in serum, plasma, and other biologic fluids are consistent with mast cell activation in systemic anaphylaxis and other immediate hypersensitivity allergic reactions [7]. Also, as an immunohistochemical stain, tryptase has proven diagnostic value in patients with mastocytosis [8]. All leukocytes, except for basophils, do not have any detectable amounts of tryptase. Even though basophils have some tryptase in their granules, tryptase quantity in basophils was described as less than 1% of those in tissue mast cells [9].

Basophils share many features with mast cells, including expression of FcεR1, secretion of Th2 cytokines, metachromatic staining, and release of histamine after activation, but they constitute a distinct lineage having many unique features [10]. Basophils are the least common of the circulating white blood cells, comprising less than 1% of the total leukocyte count. Basophilia is defined as an absolute basophil count above 0.2 x 10^3^/mm^3^, which can be seen in reactive or neoplastic conditions. Reactive basophilia is an uncommon blood finding but can be found in allergic disorders, inflammatory reactions, and certain infections [10]. In contrast, neoplastic causes are more frequently seen in clinical settings that are reactive conditions. Chronic MPNs, especially CML, are the most common entity in which an absolute basophilia is noted [2].

CML is a clonal disorder of a pluripotent stem cell that involves all myeloid lineages with a primary proliferation of granulocytes and their precursors in the blood and bone marrow [2]. The disease course is triphasic, with a progression from an initial CP followed by an AP or BP. One of the criteria to establish the diagnosis of an AP includes the presence of ≥20% basophils in the peripheral blood [1].

In our case, we had a patient with a long-standing history of CML that had progressed to an AP based on basophilia and cytogenetic analysis (trisomy 8). The significant increase of basophils was noted in the bone marrow and was confirmed by morphology and phenotypic findings based on flow and immunohistochemistry (CD123). Interestingly, tryptase stain showed significant staining of basophils, with few scattered mast cells noted with CD117 (Figures 1A-1F).

Some studies in the literature had previously reported that immature neoplastic basophils can express a significant amounts of tryptase. However, based on our search, there were only two studies that analyzed that possibility and supported it [11,12]. Samorapoompichit et al. demonstrated that the basophils obtained from patients with CML, idiopathic myelofibrosis, and myelodysplastic syndrome showed reactivity with tryptase on immunoelectron microscopy. In contrast, no significant amount of tryptase was detected in normal basophils [11]. Horny et al. demonstrated that basophils in two cases of CML expressed tryptase [12].

## Conclusions

Our case findings are similar to the previous two studies and suggest that tryptase should not be regarded as a specific marker for mast cells, especially in myeloid neoplasms (including CML). Further studies are required to evaluate the presence of tryptase expressing basophils in myeloid neoplasms. Another question that can be raised is whether this can be used as a marker to monitor some of these myeloid neoplasms.

## Appendices

Follow-up: The patient was matched for unrelated donor peripheral blood stem cell transplant. In the interim, several bone marrow biopsies have been performed which did not reveal any evidence of residual disease. The BCR/ABL1 has been undetectable by cytogenetic analysis and polymerase chain reaction (PCR). Twelve months’ post-transplant follow-up showed that the patient is doing well.
